# Spatial optimization of vaccine supply networks in complex terrains: balancing economic feasibility and public health equity

**DOI:** 10.3389/fpubh.2026.1971221

**Published:** 2026-10-30

**Authors:** Jiajun Liao, Weijian Zhang, Zhao Zhang, Xiaorong Duan, Yu Wang, Shiping Wang, Lin Gao, Yonghong Huang

**Affiliations:** 1 School of Logistics, Chengdu University of Information Technology, Chengdu, China; 2 Xichang People's Hospital, Xichang, China; 3 College of Management Science, Chengdu University of Technology, Chengdu, China; 4 Chengdu Jinniu District School Safety Emergency Guidance Center (Chengdu Jinniu District Community Education College), Chengdu, China; 5 Xichang Center for Disease Control and Prevention, Xichang, China; 6 Xichang Beicheng Community Healthcare Center, Xichang, China; 7 Xichang Second People's Hospital, Xichang, China

**Keywords:** accessibility, mountainous regions, multi-objective optimization, resilience, spatial equity, vaccine supply network

## Abstract

**Background:**

Vaccine supply planning in hazard-prone terrains faces compounding geographic, socioeconomic, and operational barriers. Conventional models relying on planar distances and static metrics often fail to capture true accessibility disparities, often driving inefficient infrastructure allocation and exacerbating health inequities.

**Purpose:**

This study develops an integrated decision-making framework coupling spatial equity, economic feasibility, and multi-scenario resilience to systematically reconfigure the vaccine supply network across 183 counties in Sichuan, China.

**Methods:**

Using high-resolution road network data from the AMap Application Programming Interface (API), we model actual geographic impedance based on travel time. An adapted two-step floating catchment area (2SFCA) method, embedded with socioeconomic vulnerability indicators, pinpoints baseline accessibility disparities. Candidate hubs are selected via entropy-weighted Technique for Order Preference by Similarity to Ideal Solution (TOPSIS) combined with spatial-equity quotas, and a candidate-pool capacity-sufficiency check (α coefficient) determines the final candidate-pool size. A multi-objective mixed-integer linear programming (MO-MILP) model then co-optimizes hub configuration, capacity scaling, and cross-regional dispatch, endogenously balancing operational efficiency, equity penalties, and capacity expansion costs.

**Results:**

Under entropy-weighted TOPSIS ranking with spatial-equity quotas, the initial 61-hub pool (including 21 existing CDC anchors) provides aggregate capacity equal to 82% of total demand. The capacity-sufficiency rule therefore expands the pool to C* = 103 hubs that satisfy Cap(C*) ≥ 1.2 × total demand. The optimized baseline network (103 candidate hubs, 99 with positive flows) achieves zero unmet demand through endogenous capacity expansion and single-source assignment, with a total objective value of 18,510,357. Sensitivity analysis across α = 1.0–1.4 shows that higher sufficiency coefficients increase the candidate pool (76 → 150 hubs) while reducing required capacity expansion by 63%. Fixed-topology resilience tests indicate zero shortages under all structural failure scenarios when emergency expansion is permitted, whereas zero-expansion stress tests bound shortage-free surge tolerance between +10% and +20% demand growth.

**Conclusion:**

Integrating spatial equity and capacity sufficiency at the candidate-pool stage limits the risk that pool-level capacity deficits are later compensated through node-level expansion. The α coefficient serves as a planning parameter for balancing network redundancy and expansion needs. The α = 1.2 configuration provides a robust and equitable vaccine supply network for mountainous regions under the model’s cost assumptions.

## 1 Introduction

Immunization programs remain a cornerstone of global public health. By reducing the burden of vaccine-preventable diseases and promoting health equity, they play a pivotal role in improving population health and maintaining socioeconomic stability (1, 2). However, achieving immunization targets relies not only on effective vaccines and vaccination strategies but also on stable and efficient vaccine supply networks. Vaccine storage, transportation, and distribution are highly constrained by geographical conditions, infrastructure availability, and operational capabilities. Consequently, the spatial configuration of these networks directly determines vaccine accessibility, safety, and the overall effectiveness of immunization interventions (3, 4).

Regions characterized by complex terrains and frequent natural disasters, such as Sichuan Province, face substantial challenges in establishing resilient vaccine supply networks. Spatial isolation and limited connectivity severely hinder the delivery of essential health resources to remote communities. Traditional efficiency-oriented logistics models often present inherent contradictions: on one hand, minimizing transport costs tends to concentrate service resources in high-demand areas, thereby widening the accessibility gap between urban and rural areas in mountainous regions (5, 6). On the other hand, public health networks possess distinct social attributes and cannot be designed solely based on economic efficiency (7). Furthermore, relying on large-scale infrastructure expansion to improve coverage in sparsely populated areas imposes heavy long-term fiscal burdens (8). Compounding these challenges, frequent natural disasters in mountainous regions disrupt transport corridors and distribution nodes, further testing the network’s resilience to sudden disruptions.

Although existing research has accumulated rich theories and methods for logistics network optimization and spatial accessibility assessment in public health, integrated planning frameworks that simultaneously balance geographic constraints, service equity, economic costs, and disaster resilience remain scarce. For rugged, unevenly developed mountainous environments, there is an urgent need to develop comprehensive decision-support tools to guide the spatial layout and resource allocation of vaccine distribution facilities, ultimately fostering the synergistic development of routine operational efficiency, population service equity, and system resilience against emergencies.

## 2 Literature review

### 2.1 Vaccine supply network optimization considering cost–equity trade-offs

Vaccine supply networks are fundamental to the effective implementation of immunization programs. Unlike conventional commercial goods, vaccines possess strong public good characteristics, strict quality requirements, and high time sensitivity. Their storage, transportation, and distribution processes rely heavily on stable supply infrastructure and sustained operational investment. Previous studies have demonstrated that robust supply chains are essential for improving vaccine accessibility and expanding immunization coverage. Conversely, transportation disruptions and temperature control failures may result in resource losses and undermine the effectiveness of immunization services (1–4).

A large number of mathematical programming approaches have been developed for vaccine supply chain planning, including facility location, routing optimization, and resource allocation. As summarized by Lemmens et al. (9), vaccine supply chain optimization has gradually evolved from single-objective cost minimization toward multi-dimensional decision-making involving facility configuration, transportation coordination, and supply capacity management. However, existing models have not yet fully addressed the trade-off between economic efficiency and public service equity, although multi-objective vaccine location models that couple equity with health outcomes have recently emerged (10).

Cost-oriented optimization models may concentrate resources in high-demand areas, potentially widening disparities in immunization accessibility across geographically complex regions. Conversely, some coverage-oriented strategies emphasize accessibility improvement while paying insufficient attention to long-term infrastructure investment and operational sustainability. Therefore, integrating spatial accessibility and health equity into vaccine supply network optimization while maintaining economic sustainability remains a critical challenge for public health resource allocation in complex terrains.

### 2.2 Spatial accessibility and health equity

Spatial accessibility is a central measure of equity in public health service delivery. Evidence shows that effective access to care depends not merely on resource availability but on the interplay of geographic topology, transport impedance, and socioeconomic conditions (11, 12). In mountainous terrain with pronounced relief, physical distance can be amplified by nonlinear friction along transportation corridors. Jamtsho et al. (11) and Fonseca et al. (12) demonstrated that the combination of natural geographic barriers and uneven health resource distribution can produce ‘spatial deprivation’ in remote areas. Beyond accessibility, service network design also hinges on adequate aggregate capacity: healthcare capacity planning is a well-established field addressing the prospective balancing of supply capacity against demand (13, 14), reliable capacity forecasting is recognized as foundational to service continuity (15), and aggregate-capacity constraints have been formalized in facility location research (16).

In immunization programs, such spatial heterogeneity may translate into unequal health outcomes. Zero-dose children are disproportionately concentrated in areas where geographic remoteness, socioeconomic disadvantage, and limited health service capacity overlap, indicating that the combined effects of poor physical accessibility and social vulnerability substantially constrain equitable vaccine access (17–19). Therefore, vaccine supply-chain design should not rely solely on macro-level coverage indicators. It must also address fine-grained spatial service disparities so that efficiency-oriented planning does not systematically overlook geographically disadvantaged populations.

Measurement methodology has progressed. Recent work replaces Euclidean distance with road-network travel time (20, 21) and begins to incorporate socioeconomic attributes into accessibility indices (22, 23). Yet most studies remain confined to post-hoc, static diagnosis. Supply-chain optimization models rarely treat multidimensional regional vulnerability or real terrain impedance as endogenous decision variables embedded directly in network redesign. Unifying spatial friction, economic vulnerability, and equity objectives within a single quantitative framework remains a key challenge for improving immunization accessibility in complex geographic settings.

### 2.3 Resilient vaccine supply chain

Recent natural disasters, public health emergencies, and growing global supply-chain uncertainties have made resilience a central concern in vaccine network research. Conventional design typically prioritizes cost reduction and operational efficiency, yet highly centralized structures may compromise system stability when sudden shocks occur. Supply-chain resilience theory holds that network design should go beyond routine efficiency, securing the capacity to maintain critical service continuity and achieve orderly recovery under disruption.

Dolgui et al. introduced the ‘Ripple Effect,’ showing that localized node failures or supply interruptions can propagate upstream and downstream through network topologies, degrading overall performance and impeding recovery (24). In the context of Coronavirus Disease 2019 (COVID-19), Ivanov demonstrated via simulation that public health emergencies substantially impair global supply-chain stability, and that structural features and redundancy levels are key determinants of systemic shock resistance (25). Building on this, Ivanov and Dolgui proposed the concept of ‘viability,’ arguing that supply-chain design should move beyond static cost minimization toward long-term continuity in uncertain environments through network reconfiguration, multi-node coordination, and dynamic adaptation (26).

Unlike commercial supply chains, disruptions in the vaccine supply chain directly threaten population-level immunity, and their consequences cannot be measured by financial losses alone. Because vaccine delivery underpins public health, node failures, road closures, or demand surges may not only reduce operational efficiency but also limit access to immunization services in remote and vulnerable regions. Vaccine supply-network planning must therefore balance economic feasibility, service equity, and systemic resilience. Yet existing literature remains largely confined to cost minimization, topological analysis, or resilience evaluation under single-disruption scenarios. Systematic optimization frameworks that jointly account for real transport impedance, regional socioeconomic disparities, and equity constraints in complex geographic environments remain scarce.

### 2.4 Research gaps and contributions

Existing research provides a theoretical foundation for vaccine supply network planning and supply chain resilience modeling. However, in complex mountainous environments, current optimization methods still face multiple limitations:

1. Realistic transport impedance and equity objectives are rarely integrated into the same optimization framework. Existing models often rely on idealized distance assumptions and therefore fail to capture the actual transport impedance of rugged terrain, potentially biasing accessibility estimates for remote areas. Socioeconomic vulnerability is also more often used for ex post evaluation than embedded as an equity constraint in facility location decisions.
2. Candidate hub pools are typically generated based on suitability or spatial equity criteria alone, without explicitly constraining their aggregate supply capacity. An undersized candidate pool may shift supply deficits to downstream node-level capacity expansion, thereby restricting the design space at the planning stage.
3. Disruption scenarios remain insufficiently addressed. Existing network designs rarely incorporate node failures, demand surges, and their compound effects in a systematic manner, limiting their ability to assess network performance under abnormal conditions.

To address these gaps, this study develops an optimization framework for vaccine supply networks in mountainous Sichuan Province. The main contributions are threefold. First, real-road-network impedance and socioeconomic vulnerability are incorporated to jointly optimize operational efficiency and spatial equity. Second, an α-based capacity sufficiency criterion is introduced to guide candidate-pool generation alongside TOPSIS ranking and spatial equity considerations. Third, a fixed-topology stress-testing framework quantifies network resilience under node failures, demand surges, and compound disruptions, while α-sensitivity analysis informs capacity and redundancy planning.

## 3 Theoretical framework

### 3.1 Spatial justice theory and equitable vaccine accessibility

The theory of spatial justice posits that geographic space actively shapes the distribution of public resources and social advantages (27). In complex mountainous terrains, geographic isolation inherently exacerbates socioeconomic vulnerability, often leading to unequal healthcare accessibility when resource allocation relies solely on cost minimization. From a public health perspective, addressing such structural determinants is crucial for health equity (28), suggesting that resource allocation should consider compensatory mechanisms for disadvantaged populations (29).

Guided by this perspective, our study operationalizes geographic equity into endogenous equity-related components within the optimization framework. Methodologically, measuring spatial accessibility must transcend Euclidean distances by accounting for genuine physical impedance and socioeconomic contexts (30). In the subsequent modeling phase, this theoretical imperative is translated in two ways. First, an economic vulnerability index ( $V_{i}$ ) is embedded into the adapted two-step floating catchment area (2SFCA) baseline assessment to amplify the weighted demand of disadvantaged counties. Second, a vulnerability-adjusted time penalty is integrated into the multi-objective programming objective (Equation 10). This component computationally exacts a higher toll for service delays in economically fragile regions, so that peripheral accessibility carries explicit weight alongside aggregate cost efficiency.

### 3.2 Supply chain resilience theory and adaptive capacity

While spatial justice informs equitable baseline coverage, supply chain resilience theory emphasizes the network’s survivability under uncertainty. Resilience is defined as a network’s capability to absorb, adapt to, and maintain essential functions under unexpected disruptive shocks (31). In disaster-prone environments, traditional supply chains that maximize operational efficiency through extreme node centralization may suffer from systemic fragility, where localized disruptions can paralyze the entire network (32). Specifically in public health, a resilient health system must maintain core functioning during crises, which requires deliberate structural buffers and adaptive capacity (33).

Within this framework, these resilience principles are translated into a node-level dynamic capacity expansion mechanism. Specifically, the model adaptively adjusts capacity subject to expansion penalties, thereby mitigating excessive dependence on centralized hub structures. The practical effectiveness of this resilience design is evaluated through multi-scenario stress testing, examining the network’s ability to use adaptive capacity to absorb local hub failures and demand surges while maintaining service under compound disruptions (26).

## 4 Materials and methods

The proposed framework integrates spatial accessibility assessment, capacity-sufficiency screening, and mathematical co-optimization to guide vaccine supply network design in mountainous terrains (Figure 1). The framework links geographic impedance analysis, equity-oriented hub selection, MO-MILP optimization, and resilience stress testing under demand surges and structural disruptions.

**Figure 1:**
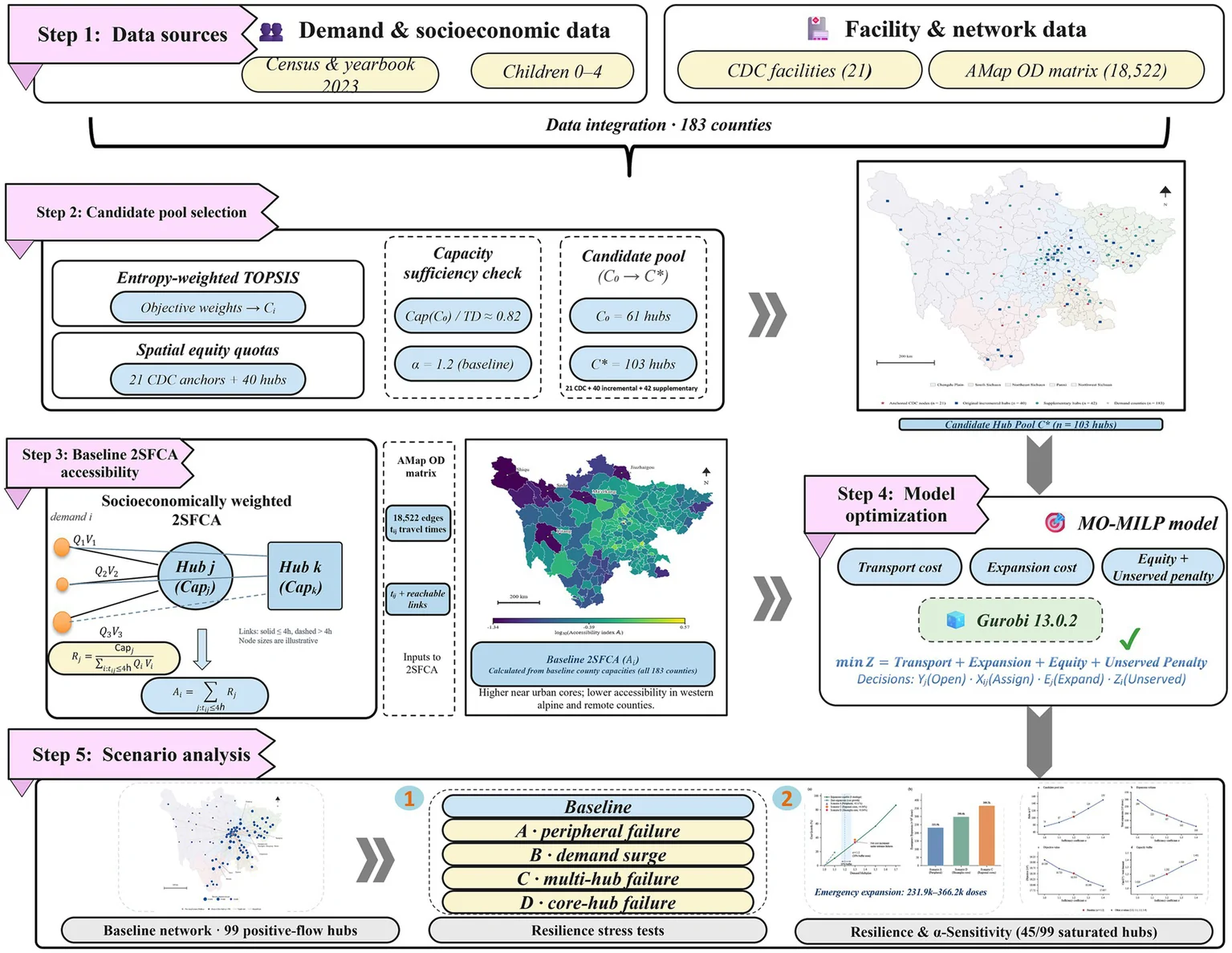
Methodological framework of the integrated vaccine supply network optimization.

### 4.1 Study area overview and construction of a multi-source heterogeneous database

This study adopts 183 counties in Sichuan Province as the fundamental spatial units of analysis. Characterized by complex topography and pronounced regional disparities in demographics and economic development, Sichuan presents significant spatial transport impedance and equity challenges for vaccine distribution. It thus serves as an ideal empirical setting for network optimization in rugged terrains.

To parameterize the optimization model, we constructed a multi-source heterogeneous database. Demographic and socioeconomic data—sourced from the Seventh National Population Census and the 2023 Sichuan Health Statistical Yearbook—incorporate total resident population, children aged 0–4, absolute and per capita Gross Domestic Product (GDP), and the number of health professionals. Spatially, county centroids were extracted via the AMap geocoding API. Using AMap’s driving-route interface, matrices for real-world road distances and travel times were generated. All spatial data processing and visualizations were executed using Python (GeoPandas) and GIS platforms, with classifications based on the Natural Breaks (Jenks) method.

### 4.2 Road-network impedance acquisition via the AMap API

The OD impedance matrix was constructed and expanded alongside the candidate hub pool, comprising 18,522 directed edges. All OD pairs were queried through a unified AMap Web Service API workflow. For each origin–destination pair, the v5 driving route service returned the road-network distance in meters and travel time in seconds between county centroids. Coordinates were converted from WGS84 to the AMap GCJ-02 reference system before routing. Each impedance value represents a directed, route-based measurement. Each edge was assigned an edge_source label to record its retrieval batch: the Original batch contains the 10,920 edges of the initial candidate pool, while AMap_Trunk and AMap_Branch contain 42 and 7,560 edges retrieved during candidate-pool expansion. This labeling provides traceability for the complete OD impedance dataset.

### 4.3 Candidate hub selection via entropy-weighted TOPSIS and spatial equity constraints

To identify candidate hubs for the vaccine supply network across 183 counties in Sichuan Province, we construct an entropy-weighted Technique for Order Preference by Similarity to Ideal Solution (TOPSIS) model using two positive indicators: the population aged 0–4 (X1), capturing immunization demand, and GDP (X2), reflecting economic capacity for vaccine infrastructure. The entropy weights were 0.3839 for X1 and 0.6161 for X2. TOPSIS ranks counties according to their relative closeness to the ideal solution and has been widely applied to healthcare facility suitability assessment.

First, the entropy weight method determines objective indicator weights. Given n=183 evaluation units and m=2 indicators, min-max normalization yields the proportion $p_{\mathrm{ij}}$ , information entropy $e_{j}$ , and weight $w_{j}$ (Equation 1):


$$e_{j}=-\frac{1}{\ln(n)}\sum_{i=1}^{n}p_{\mathrm{ij}}\ln(p_{\mathrm{ij}})$$

$$w_{j}=\frac{1-E_{j}}{\sum_{j=1}^{m}(1-E_{j})}$$ (1)

The weighted normalized matrix is constructed by multiplying each normalized indicator value by its corresponding entropy weight (Equation 2).


$$Y_{\mathrm{ij}}=w_{j}\times Z_{\mathrm{ij}}$$ (2)

From this matrix, Euclidean distances to the positive and negative ideal solutions (D^+^*ᵢ* and D^−^*ᵢ*) are computed to derive the suitability index C*ᵢ* (Equation 3), where the positive ideal solution is the column-wise best weighted-normalized value across all counties and the negative ideal the column-wise worst.


$$C_{i}=\frac{i_{-}^{D}}{i_{+}^{D}+i_{-}^{D}}$$ (3)

A higher $C_{i}$ indicates greater hub suitability.

Strictly unconstrained TOPSIS ranking tends to concentrate resources in densely populated and economically advantaged areas, which can compromise basic coverage across remote regions. To address this spatial imbalance, we introduce spatial equity constraints through a dual-track “21 + 40” hub configuration.

First, 21 existing Centers for Disease Control and Prevention (CDC) nodes are retained to anchor the baseline network. Second, 40 additional nodes are selected from the highest-ranked remaining candidates, with regional quotas allocated across Sichuan’s five economic zones using an equal weighting of population and geographic area. To prevent intra-regional clustering, the number of additional nodes assigned to each prefecture-level city is capped at one-third of its zone’s incremental quota. This spatial screening produces the initial candidate pool (C₀), comprising 61 hubs. The unified TOPSIS scores are computed across all 183 counties and subsequently utilized for regional ranking and quota-based candidate selection.

### 4.4 Candidate-Pool capacity sufficiency check

The candidate-pool generation includes a capacity sufficiency check. Motivated by capacity-adequacy principles in healthcare capacity planning (13, 14)and aggregate-capacity constraints in facility location (16), the check is evaluated at the candidate-pool level: let Cap(C) denote the aggregate baseline capacity of a candidate pool C , defined as $\mathrm{Cap}(C)=\sum_{j\in C}\mathrm{Ca}p_{j}$ , and let $\mathrm{TD}=\sum_{i\in I}Q_{i}$ denote the total province-wide monthly vaccine demand. The sufficiency condition requires that (Equation 4):


$$\mathrm{Cap}(C^{*})\geq \alpha \times \mathrm{TD}$$ (4)

where α is an exogenous planning parameter held fixed during optimization.

### 4.5 Candidate augmentation

When $\mathrm{Cap}(C_{0})<\alpha \times \mathrm{TD}$ , the initial candidate pool is expanded using the remaining unselected counties. This augmentation operates strictly monotonically: $C_{0}$ is retained in its entirety, and the five economic zones maintain balanced spatial coverage. Specifically, unselected counties within each zone are sorted by their unified TOPSIS composite scores and appended iteratively to form a supplementary set $S^{+}$ until the capacity threshold is met. The final pool is defined as $C^{*}=C_{0}\cup S^{+}$ , yielding an endogenous candidate cardinality governed directly by data-driven capacity requirements.

### 4.6 Baseline accessibility assessment using a socioeconomically weighted 2SFCA method

To evaluate baseline spatial accessibility across Sichuan Province and pinpoint underserved regions, an adapted 2SFCA method adjusted for economic vulnerability was implemented with a 4-h (14,400 s) effective service threshold (t_0_).

First, the effective supply-to-demand ratio Rj is calculated for each hub within the threshold (Equation 5):


$$R_{j}=\frac{\mathrm{Ca}p_{j}}{\sum_{i\in \{t_{\mathrm{ij}}\leq t_{0}\}}Q_{i}V_{i}}$$ (5)

where $\mathrm{Ca}p_{j}$ is the baseline capacity of hub j ; $Q_{i}$ represents the vaccine demand at area i ; $V_{i}$ denotes the regional economic vulnerability index; and $t_{\mathrm{ij}}$ is the real-world road travel time between nodes. The economic vulnerability index is dimensionless with values of at least 1: the province’s most affluent node attains 1 and economically weaker nodes attain larger values, so their weighted demand is amplified;

Second, spatial accessibility *A_i_* is computed for each demand node by aggregating accessible supply-to-demand ratios (Equation 6):


$$A_{i}=\sum_{j\in \{t_{\mathrm{ij}}\leq t_{0}\}}R_{j}$$ (6)

Where lower $A_{i}$ indicates severe accessibility deficits, flagging priority intervention areas.

### 4.7 Multi-objective network optimization model (MO-MILP)

#### 4.7.1 Parameters and decision variables

Building on the final candidate pool $C^{*}=C_{0}\cup S^{+}$ ( 103 hubs), we formulate a Multi-Objective Mixed-Integer Linear Programming (MO-MILP) model to optimize vaccine allocation across 183 demand counties. Model parameters, network topology, and allocation constraints are mapped directly to this expanded candidate pool.

The monthly routine vaccine demand $Q_{i}$ for node i is calculated from the population of children aged 0–4 and national immunization schedules (Equation 7), incorporating a 5% redundancy coefficient ( β=1.05 ) to buffer against logistics waste and operational fluctuations:


$$Q_{i}=\frac{P_{0-4,i}\times 22}{60}\times (\beta ),\forall i\in I$$ (7)

The economic vulnerability index $V_{i}$ is defined as $V_{i}=1/a_{i}$ , where $a_{i}=\mathrm{GD}P_{\mathrm{pc},i}/\max_{k\in I}(\mathrm{GD}P_{\mathrm{pc},k})\in (0,1]$ represents the normalized affluence index provided in the dataset (Equation 8). This dimensionless factor scales the penalty for serving vulnerable nodes from distant facilities.


$$V_{i}=\frac{\max_{i\in I}\text{GDPpc},i}{\text{GDPpc},i},\;\forall i\in I$$ (8)

The baseline static throughput capacity $\mathrm{Ca}p_{j}$ of candidate hub j is determined by the number of health professionals $H_{j}$ and their unit processing capacity M . Here, M is calibrated using the provincial supply–demand baseline multiplied by a 1.5 surge factor (Equation 9):


$$\begin{array}{c} M=\frac{\sum_{i\in I}Q_{i}}{\sum_{i\in I}H_{i}}\times 1.5,\\ \mathrm{Ca}p_{j}=H_{j}\times M,\forall j\in J \end{array}$$ (9)

Spatial impedance parameters, extracted from real-world road networks, include the trunk distance from the provincial central warehouse to the hub ( $j_{\text{trunk}}^{d}$ ), the branch distance from the hub to the demand node ( $ij_{\text{branch}}^{d}$ ), and the branch travel time ( $t_{\mathrm{ij}}$ ).

The decision space consists of three dimensions. Binary variables $Y_{j}$ and $X_{\mathrm{ij}}$ denote the activation status of hub j and the supply assignment from hub j to node i , respectively. Continuous variable $E_{j}\geq 0$ represents the dynamic capacity expansion for hub j . Finally, a binary slack variable $Z_{i}$ flags unfulfilled demand at node i due to extreme physical impedance, ensuring mathematical feasibility under strict constraints.

#### 4.7.2 Objective function and constraints

This study integrates physical logistics efficiency with public health equity. The comprehensive objective function Z minimizes transportation impedance, capacity expansion costs, and equity-based time penalties:


$$\begin{array}{c} \min Z=\sum_{i\in I}\sum_{j\in J}c_{1}Q_{i}(j_{\text{trunk}}^{d}+ij_{\text{branch}}^{d})X_{\mathrm{ij}}+c_{e}\sum_{j\in J}E_{j}\\ +w\sum_{i\in I}\sum_{j\in J}\frac{V_{i}Q_{i}t_{\mathrm{ij}}}{10000}X_{\mathrm{ij}}+M_{p}\sum_{i\in I}Z_{i} \end{array}$$ (10)

The first term minimizes trunk and branch transport impedance via unit cost coefficient $c_{1}$ . The second term curbs over-reliance on new infrastructure through an expansion penalty $c_{e}$ . The third term is a vulnerability-adjusted time penalty that translates delivery delays into an equity cost. By compounding travel time $t_{\mathrm{ij}}$ , demand $Q_{i}$ , and economic vulnerability $V_{i}$ —scaled in units of 10^4^ s and weighted by the equity weight w —this mechanism penalizes service delays more heavily in economically fragile and remote areas. This normalization further converts the time-based penalty into the same scale as the operational costs, ensuring dimensional consistency within the objective function. The final term employs the unserved penalty parameter ( $M_{p}$ ) to strictly penalize unserved nodes under extreme conditions. All cost coefficients are expressed in normalized model cost units (CU), a weighted monetary proxy with no specific currency implied.

This objective is subject to four core constraints. First, the single-source assignment and slack constraint ensures each demand node is served by exactly one hub (Equation 11), with $Z_{i}$ capturing any unserved state:


$$\sum_{j\in J}X_{\mathrm{ij}}+Z_{i}=1,\forall i\in I$$ (11)

Second, the logical activation constraint guarantees that only open hubs can provide services (Equation 12):


$$X_{\mathrm{ij}}\leq Y_{j},\forall i\in I,j\in J$$ (12)

Third, the capacity constraint ensures that the total demand assigned to a hub does not exceed its baseline capacity plus any dynamic expansion, preventing node overload (Equation 13):


$$\sum_{i\in I}Q_{i}\cdot X_{\mathrm{ij}}\leq \mathrm{Ca}p_{j}\cdot Y_{j}+E_{j},\forall j\in J$$ (13)

Finally, the expansion logic constraint prohibits inactive hubs from expanding:


$$E_{j}\leq M_{e}Y_{j},\forall j\in J$$ (14)

All models were implemented in Python and solved using Gurobi Optimizer 13.0.2. A relative MIP gap tolerance of $10^{-2}$ was imposed, with runtime limits of 3,600 s for the first-stage network optimization and 180 s for each resilience scenario. All α -sensitivity instances converged within the specified tolerance, yielding final relative gaps between $2.87\times 10^{-4}$ and $1.09\times 10^{-3}$ ; in particular, the baseline instance ( α=1.2 ) achieved a final relative gap of $6.6\times 10^{-4}$ .

### 4.8 Resilience assessment and multi-scenario stress testing

To maintain mathematical solvability while strictly penalizing unserved demand, the penalty parameter $M_{p}$ is set to $10^{9}$ in the baseline model and adjusted to $10^{8}$ for stress-testing. Hub–county assignments are determined over the full set of road-network-accessible links between regional hubs and demand counties, without an imposed service-radius cap; under severe disruptions, surviving hubs re-compete for assignments across this unrestricted link set. Upstream supply originates from an external provincial repository outside the modeled boundary; disruptions are thus restricted to regional and local hubs.

Five scenarios evaluate network resilience under a fixed baseline topology:

1. Baseline: Standard demand under routine operational constraints;
2. Scenario A (Peripheral Failure): Disruption of peripheral hubs in Rangtang County and Kangding City;
3. Scenario B (Demand Surge): Uniform demand surges across expansion-capable (10–70%) and zero-expansion (10–30%) configurations;
4. Scenario C (Multi-Hub Disruption): A 30% demand surge combined with the removal of three major secondary hubs (Qingyang, Xichang, Jinniu);
5. Scenario D (Core-Hub Disruption): A 30% demand surge combined with the complete failure of the primary hub (Shuangliu).

In all disruption scenarios, surviving hubs re-compete for county allocations under baseline single-sourcing and capacity constraints. While routine expansion incurs a unit capital cost $c_{e}$ , emergency expansion in the stress-testing objective is penalized at $5c_{e}$ (replacing the routine expansion term in Equation 10); under the zero-expansion setting, emergency expansion variables are fixed to zero, while the planning-stage expansion of the baseline solution (186,595 doses) is retained within the fixed hub capacities.

Finally, a subsequent sensitivity analysis verifies the model’s robustness by systematically varying the candidate-pool sufficiency coefficient α (1.0–1.4) and subjecting the fixed network topology to disruption scenarios.

## 5 Results

### 5.1 Candidate Pool and baseline accessibility

The initial candidate pool $C_{0}$ comprises 61 hubs, including 21 existing CDC nodes and 40 incremental hubs. The baseline capacity-sufficiency evaluation ( $\mathrm{Cap}(C_{0})/\mathrm{TD}\approx 0.82$ ) identifies a structural capacity deficit, triggering an augmentation procedure that incorporates 42 supplementary hubs ( $S^{+}$ ) to establish the final candidate pool $C^{*}$ of 103 hubs, successfully satisfying the threshold $\mathrm{Cap}(C^{*})\geq 1.2\times \mathrm{TD}$ (Figure 2). Sized to serve 183 demand counties, this 103-hub candidate pool ensures aggregate capacity adequacy while maintaining computational tractability for the subsequent spatial optimization.

**Figure 2:**
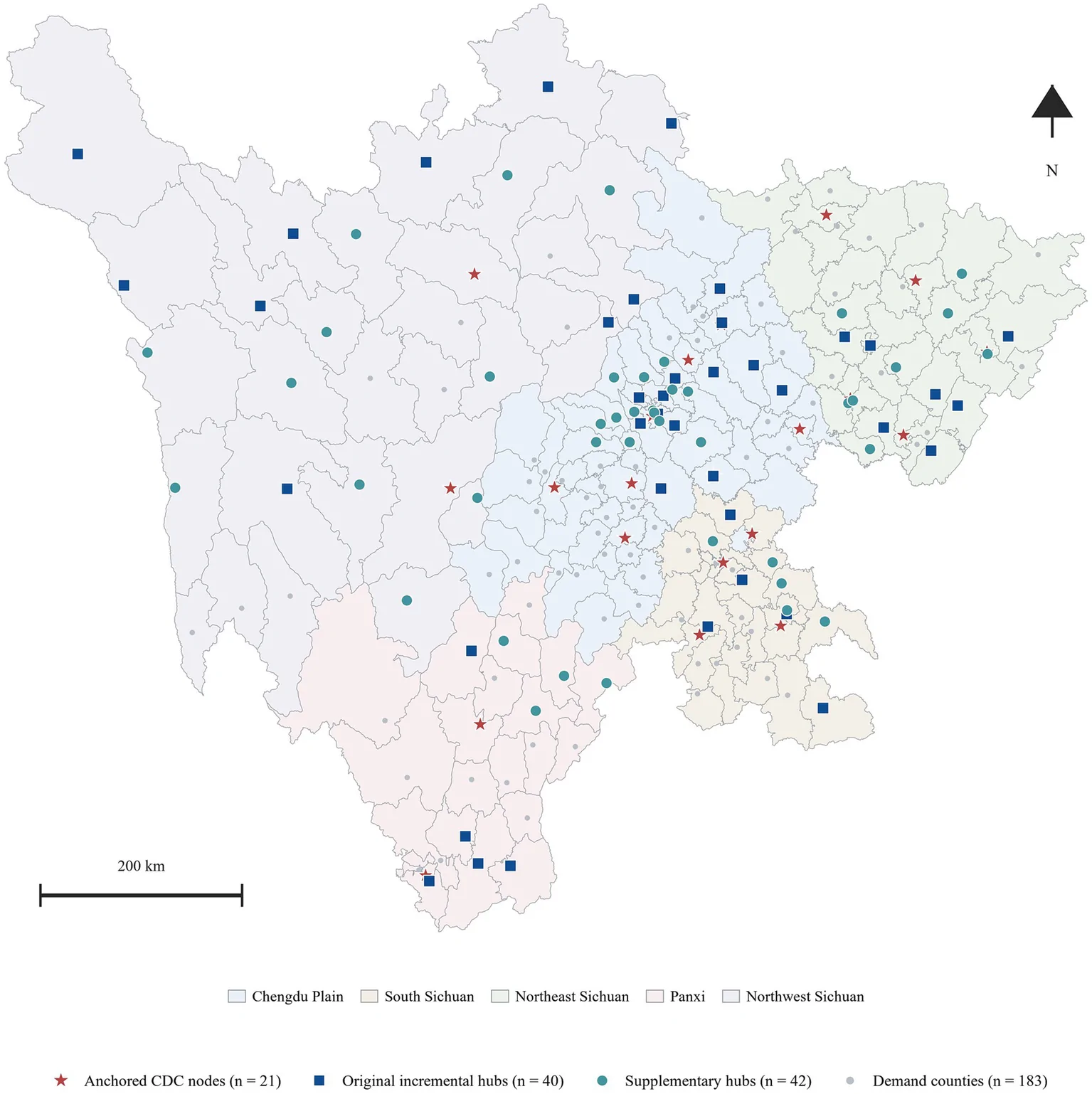
Spatial distribution of the final candidate hub pool in Sichuan Province ( $C^{*}$ , *n* = 103). Nodes are categorized into anchored CDC facilities (*n* = 21), initial incremental hubs (*n* = 40), and supplementary hubs (*n* = 42).

Baseline 2SFCA accessibility scores reveal pronounced spatial disparities across the province (Figure 3). High accessibility ( $\log_{10}A_{i}>0$ , peaking at 0.57 in Qingyang District) concentrates predominantly in the core Chengdu Plain and central urban areas. Conversely, severe accessibility deficits ( $\log_{10}A_{i}\leq -0.39$ , reaching a minimum of −1.34 ) cluster prominently in the remote, high-altitude plateau regions of western Sichuan—most acutely across Shiqu, Seda, Litang, Ma’erkang, and Jiuzhaigou. This geographical divide underscores how complex mountainous topography and dispersed rural populations constrain equitable healthcare delivery under conventional spatial allocation frameworks.

**Figure 3:**
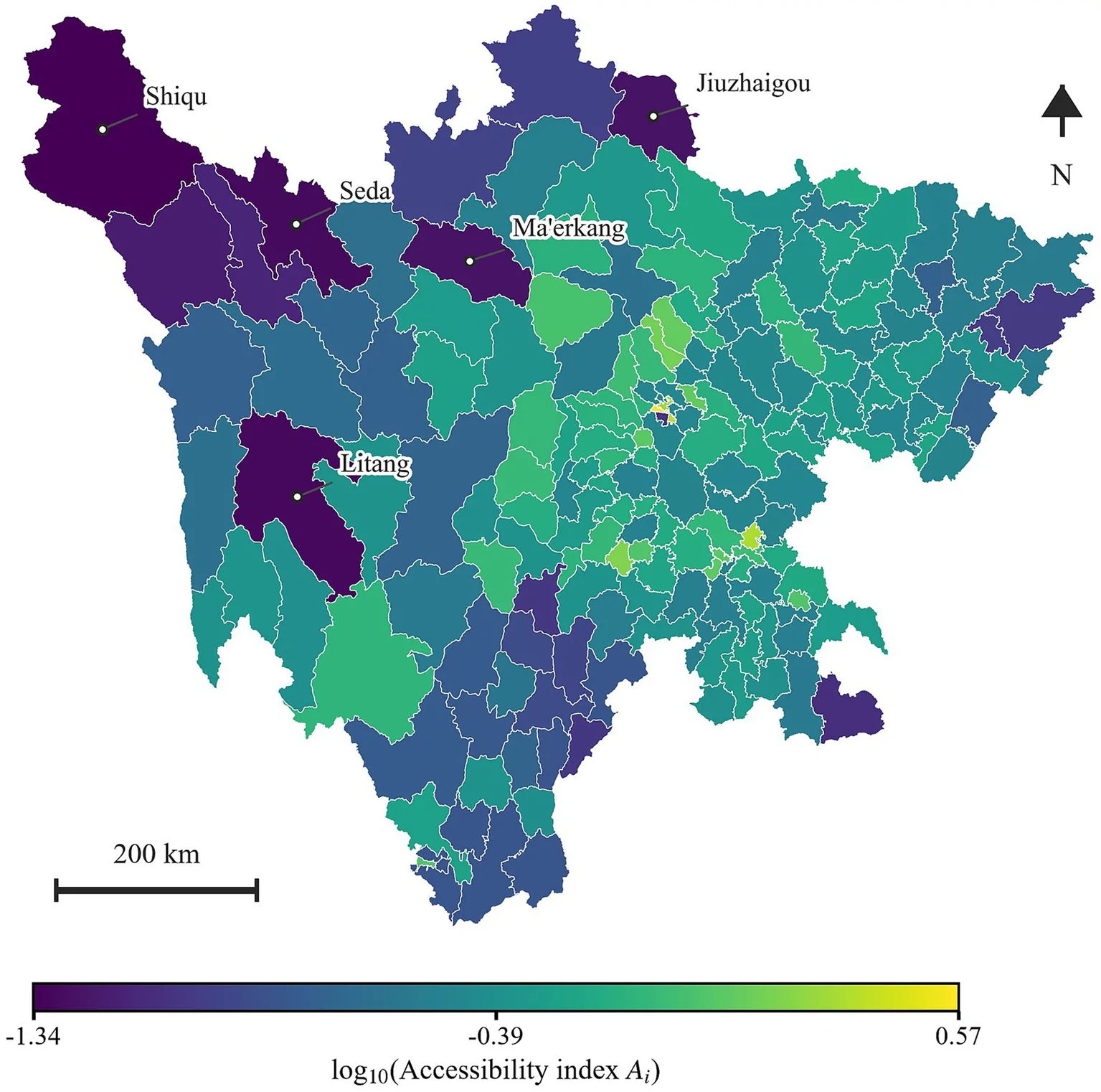
Spatial distribution of the log-transformed baseline vaccine accessibility index (*A_i_*) in Sichuan Province.

### 5.2 Hub node selection and flow assignment

Under MO-MILP optimization, the model generated a highly differentiated hub configuration (Table 1). Among the 103 candidate hubs, 99 carried positive flow. Shuangliu District handled the largest monthly dispatch volume (65,925 doses across 2 counties), followed by Qingyang (61,694 doses) and Xichang (53,367 doses). These high-throughput hubs act as regional buffers, while 4 candidate hubs entered the solution with activation variables set to one but zero assigned flow, which is cost-neutral because the formulation carries no fixed hub-opening cost.

**Table 1:** Summary of optimal capacity expansion and dispatch allocation for top 10 key hubs.

Supply hub	Covered counties	Monthly dispatch volume	Capacity expansion	Utilization
Shuangliu District	2	65,925	9,528	100.0%
Qingyang District	5	61,694	0	78.7%
Xichang City	6	53,367	17,721	100.0%
Jinniu District	2	50,708	0	85.8%
Shizhong District (Leshan)	9	47,385	16,678	100.0%
Xuzhou District	5	45,536	20,096	100.0%
Fucheng District	4	44,248	0	94.3%
Dongpo District	6	39,024	10,574	100.0%
Wuhou District	1	33,617	0	18.9%
Shunqing District	4	32,656	0	83.1%

Geographically, trunk distances from the provincial center to hubs ranged from 4.97 km (Qingyang) to 931.55 km (Shiqu). Branch distances displayed marked spatial heterogeneity: hubs serving compact local clusters (e.g., Dachuan) exhibited average service radii as low as 2.83 km, establishing dense, high-efficiency local delivery zones. Conversely, peripheral hubs (e.g., Litang and Kangding) spanned wide radial coverage (166.30 km and 100.96 km) to guarantee service in remote terrains. The average branch distance across the network was 37.11 km, with a maximum of 310.07 km (Litang to Derong). Topologically, the optimized network forms a robust two-echelon radial structure anchored by Shuangliu District, surrounded by a protective ring of high-throughput secondary hubs (Figure 4). Comprehensive routing data are available in Supplementary File.

**Figure 4:**
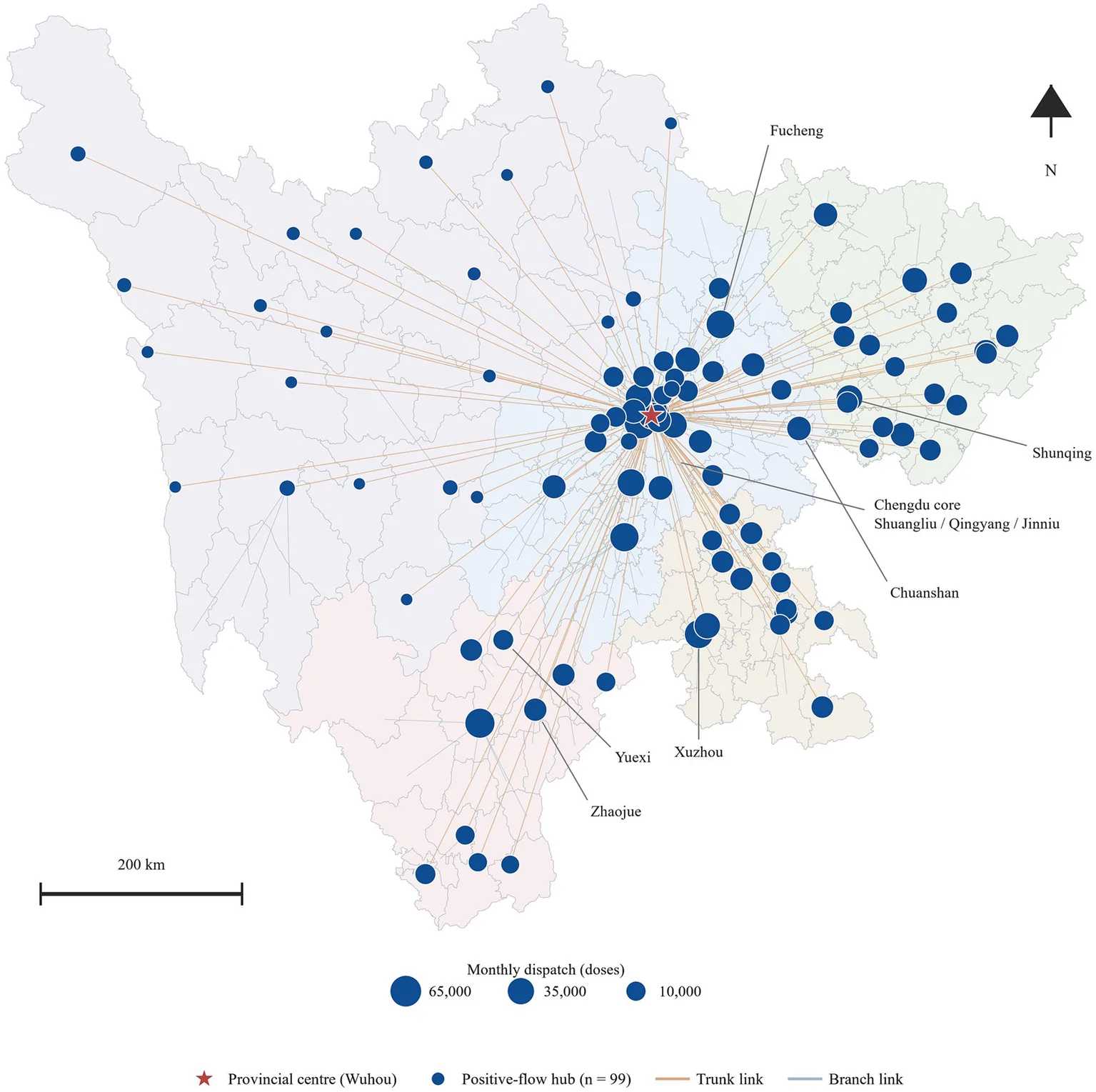
Optimal baseline distribution network configuration (*α* = 1.2). Circles denote the 99 positive-flow hubs, with marker size proportional to monthly dispatch volume.

### 5.3 System robustness and resilience assessment under multiple disruptions

Tables 2, 3 and Figure 5 summarize stress-test performance under the fixed topology, with node details in Supplementary Table 2. The Baseline achieved full demand satisfaction at a total cost of $1.851\times 10^{7}$ across 99 active hubs.

1. Scenario A (Peripheral Failure): The network exhibited strong localized robustness. Demand was smoothly rerouted to adjacent hubs with zero shortages, incurring a 3.84% cost increase under routine demand (zero-expansion) and a 33.03% increase under a +30% surge (with 231,900 doses of emergency expansion).
2. Scenario B (Demand Surge): In the expansion-capable setting, capacity scaled monotonically (58,627 to 750,700 doses from +10% to +70% surge) to avert shortages entirely. Without expansion, the physical capacity threshold was breached at +20% surge (71,308-dose shortage) and widened at +30% (203,084 doses), consistent with the ~20% safety margin ( α=1.2 ).
3. Scenarios C & D (Multi-Hub & Core-Hub Failures): Under a +30% surge, distributed rerouting fully absorbed the concurrent failures of three secondary hubs (Scenario C) or the primary Shuangliu hub (Scenario D) without shortages, requiring emergency expansions of 366,215 and 298,552 doses, respectively.

**Table 2:** System resilience under demand-increase scenarios.

Setting	Scenario	Demand multiplier	Objective *Z* (model cost units)^b^	Total Shortage (doses)^c^	Cost Growth (%)^a^
Expansion-capable	Baseline	1.0×	18,510,357	0	0.00%
Expansion-capable	Scenario B (+10% demand)	1.1×	20,446,371	0	10.46%
Expansion-capable	Scenario B (+30% demand)	1.3×	24,603,126	0	32.92%
Expansion-capable	Scenario B (+50% demand)	1.5×	28,957,877	0	56.44%
Expansion-capable	Scenario B (+70% demand)	1.7×	34,498,697	0	86.38%
Zero-expansion	Baseline	1.0×	18,417,060	0	0.00%
Zero-expansion	Scenario B (+10% demand)	1.1×	21,884,614	0	18.83%
Zero-expansion	Scenario B (+20% demand)	1.2×	124,985,289	71,308	578.64%
Zero-expansion	Scenario B (+30% demand)	1.3×	428,930,827	203,084	2,228.99%

^a^Cost growth is calculated against setting-specific baselines (*Z* = 18,510,357 for expansion-capable; *Z* = 18,417,060 for zero-expansion, net of routine expansion capital).^b^Penalty parameter *M_p_=10^8^* is levied per disconnected county irrespective of deficit volume. ^c^Demand and shortages are expressed in monthly doses. Extreme cost increases under zero-expansion 1.2× and 1.3× surges stem primarily from *M_p_* penalties rather than routing/transportation costs.

**Table 3:** System resilience under structural-failure scenarios.

Setting^c^	Scenario^d^	Demand multiplier^a^	Objective *Z* (model cost units)	Total Shortage (doses)	Cost Growth (%)^b^
Expansion-capable	Scenario A (peripheral failure)	1.3×	24,624,444	0	33.03%
Expansion-capable	Scenario C (regional multi-hub failure)	1.3×	25,352,322	0	36.96%
Expansion-capable	Scenario D (largest-hub failure)	1.3×	24,759,454	0	33.76%
Zero-expansion	Scenario A (peripheral failure)	1.0×	19,123,683	0	3.84%
Zero-expansion	Scenario C (regional multi-hub failure)	1.0×	19,911,965	0	8.12%
Zero-expansion	Scenario D (largest-hub failure)	1.0×	19,156,634	0	4.02%

^a^Demand configurations: Expansion-capable Scenarios A, C, and D apply 1.3× surge demand; corresponding zero-expansion scenarios use nominal (1.0×) demand ^b^Cost growth is calculated relative to the setting-specific baselines from Table 2. ^c^Fixed-topology tests keep baseline activations fixed, exclude failed hubs, and re-solve county allocation among surviving operational hubs. ^d^Disrupted hub locations and emergency expansion quantities are cataloged in Supplementary Table 2.

**Figure 5:**
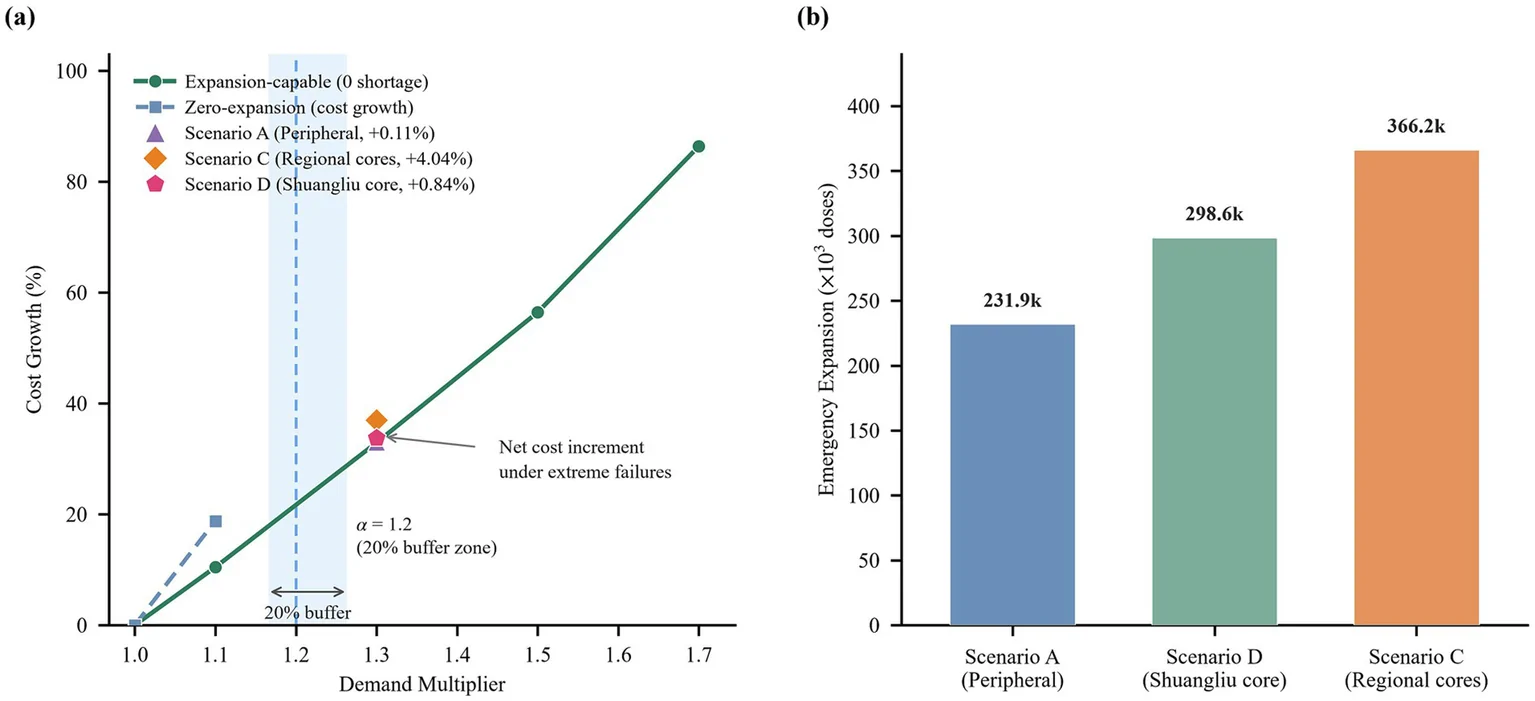
Quantitative resilience assessment and stress testing of the optimized network: **(a)** Cost growth versus demand multiplier under the expansion-capable and zero-expansion settings, with the three failure scenarios superimposed; **(b)** emergency expansion volume by failure scenario.

### 5.4 α-Sensitivity of the candidate-pool sufficiency coefficient

To examine how candidate-pool capacity sufficiency shapes network performance, α was evaluated across 1.0 , 1.1 , 1.2 , 1.3 , and 1.4 , with each setting generating a distinct candidate pool $C^{*}(\alpha )$ for the MO-MILP optimization (Table 4). Increasing α systematically expanded the candidate pool from 76 to 150 hubs, raising the capacity-to-demand ratio from 1.029 to 1.401 .

**Table 4:** *α*-sensitivity of the candidate-pool sufficiency coefficient on network optimization.

*α*	C* hubs	S + hubs	Cap/TD	Economic cost (CU)	Equity penalty (CU)	Objective (CU)	Expansion (doses)	Avg. distance (km)	Flow hubs
1.0	76	15	1.029	17,250,585	1,710,408	19,109,154	296,322	54.38	73
1.1	87	26	1.114	17,206,396	1,404,626	18,722,592	223,141	46.97	84
1.2†	103	42	1.202	17,174,929	1,242,131	18,510,357	186,595	37.11	99
1.3	126	65	1.309	17,122,680	973,417	18,167,607	143,018	26.16	119
1.4	150	89	1.401	17,112,386	769,697	17,936,589	109,010	14.28	140

^†^ denotes the selected baseline (*α* = 1.2). Economic cost excludes the expansion term; Objective = Economic cost + Equity penalty + *cₑ* × Expansion.

This expansion directly influenced network configuration and cost components: total capacity expansion decreased by 63% (from 296,322 to 109,010 doses), transport cost declined by 0.80% , the equity penalty dropped by 55.0% , and the overall objective value decreased by 6.14% . Concurrently, average branch delivery distance decreased from 54.38km to 14.28km , while the number of positive-flow hubs expanded from 73 to 140.

Candidate-pool scalability analysis demonstrates that increasing α provides substantial early efficiency gains (an objective reduction of 35,142 per candidate node from α:1.0→1.1 ) to 9,626 ( α:1.3→1.4 ), while balancing computational tractability and capacity buffer beyond α=1.2 (Figure 6). Accordingly, α=1.2 establishes a balanced operational baseline by providing a ∼20% aggregate capacity buffer. This configuration captures primary expansion relief without incurring excessive network fragmentation. α=1.2 serves as an effective engineering threshold where further candidate expansion yields marginal performance improvements against growing network dispersion.

**Figure 6:**
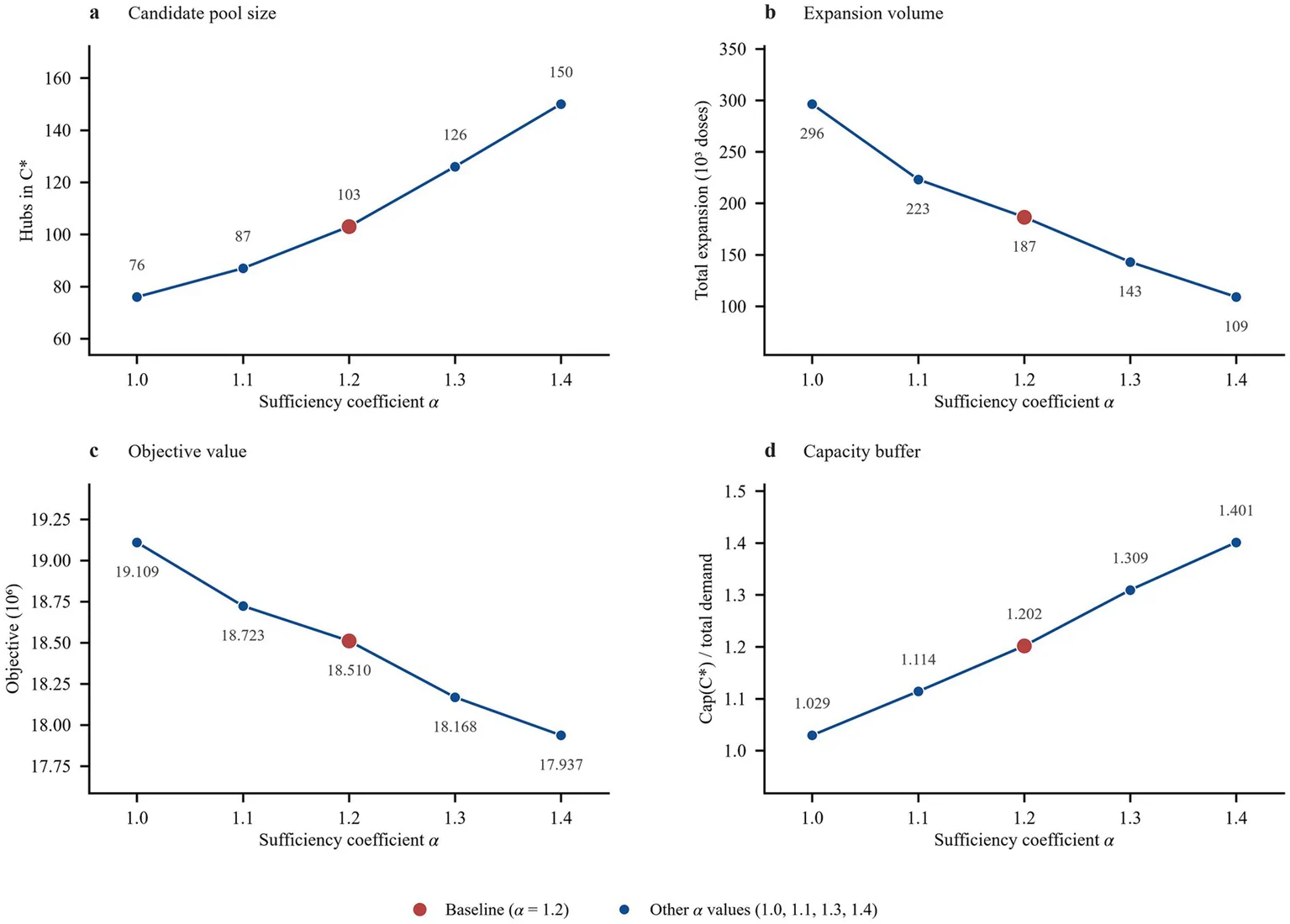
Sensitivity of network optimization to the candidate-pool sufficiency coefficient (*α*): **(a)** Candidate pool size; **(b)** total capacity expansion volume; **(c)** total objective value; **(d)** capacity buffer Cap(C*)/total demand, each versus the sufficiency coefficient *α*, with the *α* = 1.2 baseline highlighted in red.

### 5.5 Model-variant comparison: equity weights and candidate-pool design

To evaluate the operational impact of the equity objective and candidate-pool expansion, the network was re-optimized under three comparative model variants: (i) a cost-only formulation ( w=0 ) excluding the vulnerability-weighted travel time penalty; (ii) equity-weight variations ( w=0.5 and w=2 ); and (iii) a CDC-only candidate configuration restricted exclusively to the 21 existing municipal CDC facilities. All formulations maintain uniform operational parameters, baseline demand structures, and algorithmic configurations identical to the baseline benchmark ( w=1 ).

Restricting facility candidates to existing CDC infrastructure increases the total objective to 23,348,095 ( +26.1% over baseline), requires 1,050,760 doses of capacity expansion ( 5.6× baseline volume), and extends the average branch distribution distance to 106.78km (Table 5). These performance differentials confirm that augmenting the candidate network to 103 hubs substantially alleviates routing friction and local capacity bottlenecks across complex mountainous terrain.

**Table 5:** Optimization performance across model configurations: economic-equity tradeoffs and candidate pool restrictions.

Variant^c^	Economic cost (CU)^b^	Equity penalty (CU)	Positive-flow hubs	Avg. branch (km)	Max. branch (km)	Objective *Z* (CU)^a^
Cost-only (*w* = 0)	17,123,232	2,897,105	83	118.83	786.65	17,123,232
*w* = 0.5	17,218,679	1,362,224	96	44.00	310.07	17,899,791
Baseline (*w* = 1)^†^	17,268,227	1,242,131	99	37.11	310.07	18,510,357
*w* = 2	17,390,672	1,146,064	99	31.77	310.07	19,682,800
CDC-only (21 hubs)	20,340,214	3,007,882	21	106.78	665.84	23,348,095

^a^Objective formulation: *Z* = economic cost + *w* × equity penalty, evaluated under each variant’s specific equity weight *W^b^*. ^b^Cost units: Values are expressed in dimensionless cost units (CU; non-denominated). Economic cost in this table includes the planning-stage expansion cost (*cₑ* × Expansion), whereas Table 4 reports it separately. ^c^Rounding: Independent rounding of tabulated components may yield a ±1 unit difference from the reported total *Z* (e.g., 18,510,358 vs. 18,510,357 for the baseline). ^†^denotes the baseline instance.

Sensitivity to equity prioritization reveals that the equity penalty decreases monotonically as w increases—falling from 2,897,105 at w=0 to 1,146,064 at w=2 —alongside a modest rise in direct operational costs (from 17,123,232 to 17,390,672 ). Specifically, the baseline parameterization ( w=1 ) reduces the equity penalty by 57% relative to the cost-only configuration at a 0.8% economic cost premium, while simultaneously compressing the average delivery branch distance from 118.83km to 37.11km (Table 5).

### 5.6 Sensitivity analysis of key parameters

Beyond the α -sensitivity analysis, we examined the structural sensitivity of network performance to disruption factors and capacity-allocation properties:

1. Structural versus Operational Drivers: Under the expansion-capable setting, hub failures are costlier than an equivalent demand surge (33.0–37.0% versus 32.9% objective escalation at the 30% level), whereas the zero-expansion setting reverses this asymmetry—demand surges beyond 20% trigger substantial shortfalls while hub failures are absorbed through re-assignment without unmet demand (Tables 2, 3). Network performance is thus governed by the interaction between topology and capacity flexibility rather than by either factor alone.
2. Capacity Utilization Dynamics: Utilization varies considerably across the 99 positive-flow hubs, with 45 hubs operating at full capacity (Figure 7). The province-wide ∼20% capacity buffer is concentrated within a subset of peripheral facilities, indicating that targeted, location-specific redundancy enhances resilience more effectively than uniform capacity expansion.
3. Candidate-Pool Scalability: Within a ±20% variation around the candidate-pool design threshold, scaling beyond α=1.2 yields marginal performance gains while maintaining balanced candidate pool size and computational feasibility (Figure 6), supporting α=1.2 as an effective structural parameter.

**Figure 7:**
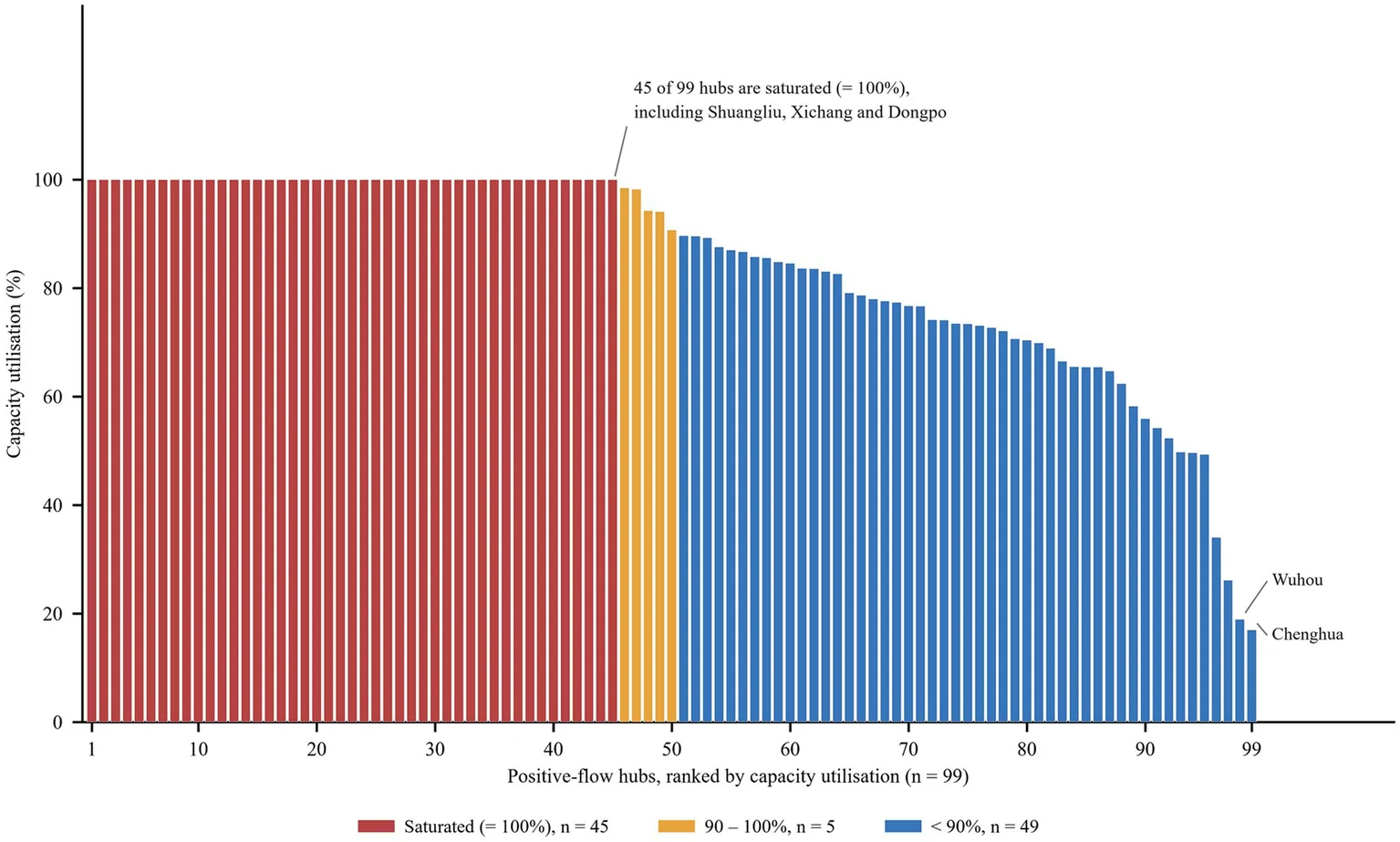
Capacity utilization of the 99 positive-flow hubs in the *α* = 1.2 baseline network.

### 5.7 Capacity utilization and core-hub fragility

To analyze the structural determinants of network resilience, capacity utilization was evaluated across all 99 positive-flow hubs (Figure 7). Hub utilization averages 84.6% (median 90.7% ), with 50 hubs exceeding 90% utilization and 45 operating at full capacity ( 100% ). Capacity redundancy exhibits strong spatial heterogeneity: major throughput facilities—including Shuangliu, Xichang, Shizhong, and Xuzhou—operate at their upper capacity bounds, while reserve buffers reside primarily within lower-throughput peripheral nodes.

Consequently, disruptions to fully loaded facilities—such as the regional hub failures in Scenario C and the core hub disruption in Scenario D—induce significant emergency expansion ( 366,215 and 298,552 doses, respectively) as displaced flows are reallocated to adjacent high-utilization nodes. This dynamic indicates that the spatial concentration of flow among key core facilities, more than aggregate province-wide capacity limits, governs network vulnerability.

### 5.8 Distributional outcomes across economic zones

Table 6 disaggregates the baseline outcomes across Sichuan’s five economic zones. All zones achieve full demand fulfillment; the longest average branch distances occur in Panxi (54.02 km) and the Northwest Ecological Demonstration Zone (46.12 km), reflecting terrain conditions. The equity penalty is largest in the Chengdu Plain (384,898) owing to its demand volume, whereas the Northwest zone accounts for only 55,842 despite its high vulnerability weighting, reflecting the dense local coverage provided by its positive-flow hubs.

**Table 6:** Distributional outcomes of the α = 1.2 baseline network across the five economic zones of Sichuan.

Economic zone	Served counties	Demand (doses)	Dispatch (doses)	Fulfillment	Equity penalty (CU)	Avg. branch (km)	Max. branch (km)
Northeast Sichuan	34	330,282	330,282	100%	276,495	26.73	170.02
Southern Sichuan	28	255,377	255,377	100%	186,207	28.02	147.56
Northwest Ecological Demonstration Zone	31	47,029	47,029	100%	55,842	46.12	310.07
Chengdu Plain	68	754,717	754,717	100%	384,898	36.46	159.65
Panxi	22	173,026	173,026	100%	338,690	54.02	220.88

## 6 Discussion

### 6.1 Spatial polarization in baseline accessibility

The baseline evaluation highlights a pronounced core–periphery polarization in Sichuan’s vaccine distribution: high accessibility is densely concentrated in the Chengdu Plain, whereas the Northwest Plateau and Panxi mountainous regions face severe service deficits. In such complex terrains, accessibility is constrained not merely by resource scale, but by the compounding effects of physical traffic friction, population sparsity, and regional underdevelopment (34, 35). By integrating the economic vulnerability index ( $V_{i}$ ), this study reveals that remote counties endure a dual burden: extreme geographic impedance and limited grassroots health infrastructure (36, 37). Methodologically, incorporating real road-network impedance alongside a socioeconomic vulnerability correction mechanism overcomes the distortions of traditional Euclidean distance models—which fail to capture the realities of winding mountain roads and elevation changes (38, 39)—thereby providing a more realistic spatial foundation for equitable network opt.

### 6.2 Operationalizing spatial justice in optimization

Conventional medical supply chain models predominantly focus on single-objective cost minimization, which inadvertently concentrates facilities in advantaged urban centers and marginalizes geographically vulnerable populations (40, 41). By incorporating dual-track candidate filtering and vulnerability-adjusted penalties, the proposed MO-MILP framework provides a quantitative pathway for operationalizing spatial justice. This approach helps transform health equity from a conventional ex-post evaluation metric into an endogenous decision-making objective within the optimization model (42–44). The optimization results indicate that actively maintaining essential distribution nodes in remote regions prevents vulnerable areas from being marginalized without severely compromising overall network efficiency. Ultimately, this indicates that achieving public health equity and resource allocation efficiency are not mutually exclusive, but can be coordinated through rational node deployment and cross-regional coordination under limited resource constraints.

### 6.3 Evaluating supply chain resilience and redundancy

Stress-testing the fixed-topology network across Scenarios A–D indicates substantial adaptive capacity. Under baseline demand conditions, the network absorbs peripheral, regional-core, and system-core hub failures without supply deficits, incurring modest cost increases of 3.84%−8.12% under the zero-expansion setting. This performance suggests that the 99 positive-flow hubs provide sufficient topological redundancy to withstand severe structural disruptions. Under uniform demand surges, the expansion-capable configuration prevents shortages up to a +70% increase via operational capacity adjustments, whereas shortage tolerance in the zero-expansion setting is bounded between +10% and +20% demand growth, directly tracing the boundary of the ∼20% safety buffer governed by α=1.2 .

These empirical stress tests corroborate core supply chain resilience principles: strategic reserve capacity and distributed facility configurations serve as functional operational buffers against correlated disruptions (45, 46). The significant emergency expansion triggered by disruptions to the primary core hub in Shuangliu ( 298,552 doses) and regional secondary centers in Qingyang, Xichang, and Jinniu ( 366,215 doses) identifies high-throughput centralized nodes as the system’s principal structural vulnerability. Consequently, vaccine distribution architectures in topographically complex environments require systematic co-optimization between operational centralization and spatial redundancy.

### 6.4 Comparison with previous studies

While spatial accessibility literature widely employs two-step floating catchment area (2SFCA) methods and their extensions for post-hoc regional assessments (47, 48), these frameworks primarily function as diagnostic tools. Even when incorporating socioeconomic indicators, they remain decoupled from proactive facility location and supply allocation. In contrast, this study embeds an economic vulnerability-adjusted index ( $V_{i}$ ) directly into the optimization objective, integrating health equity as an active structural driver of two-echelon vaccine distribution.

Closely aligned with this objective, Zhang et al. (10) developed a multi-objective MILP coupling health equity, epidemic dynamics, and economic cost for vaccine site selection. The present framework advances this line of inquiry along three distinct dimensions:

1. Spatial equity is operationalized via a vulnerability-weighted travel time penalty across a multi-tier distribution network;
2. An algorithmic capacity-sufficiency criterion ( α ) endogenously structures the candidate pool prior to mathematical optimization, preventing structural undersupply; and
3. The resulting topology undergoes rigorous multi-scenario stress testing across real-world mountain road networks under simultaneous facility outages and demand surges.

Furthermore, traditional location-routing formulations frequently approximate transit impedance using Euclidean metrics or planar road networks (49–52), which overlook the nonlinear transport resistance characteristic of complex topography. In mountainous environments, factors such as elevation gradients, high road sinuosity, and variable travel speeds substantially distort actual delivery logistics. Addressing the geographic realities of southwestern China, our approach integrates an empirical road network impedance surface directly into the optimization framework, explicitly accounting for terrain constraints and routing delays.

Finally, supply chain optimization typically treats routine operational efficiency and emergency resilience as independent modeling domains (53, 54). The proposed MO-MILP framework bridges this operational divide by combining routine distribution objectives with big- M disruption penalties and endogenous capacity redundancy. By optimizing standard logistics flows while embedding structural rerouting pathways to absorb severe node failures and demand shocks, the model seeks a balanced trade-off among economic efficiency, health equity, and systemic resilience.

### 6.5 Policy implications

These findings provide several practical implications for vaccine supply network planning in complex terrains.

First, regional coordination mechanisms should be strengthened to develop a distributed and multi-tiered network structure. Rather than relying solely on isolated county-level deployments or excessive concentration at a limited number of hubs, policymakers should enhance the role of secondary regional hubs. Scenario C demonstrates that a distributed structure can dynamically redistribute supply capacity and maintain immunization coverage under the failure of regional secondary cores.

Second, infrastructure investment should shift from uniform expansion toward spatially differentiated deployment strategies. High-demand regions may prioritize upgrading existing facilities to improve resource utilization efficiency, whereas remote mountainous areas may benefit more from lightweight decentralized service nodes and flexible delivery mechanisms due to topological constraints and diminishing returns of capacity expansion.

Third, reducing excessive dependence on single high-throughput hub nodes is critical for improving network resilience. Scenario D reveals that the failure of the largest hub (Shuangliu) triggers substantial emergency expansion (298,552 doses, second only to the multi-hub regional failure). Therefore, establishing backup supply capacity and alternative coordination mechanisms at the provincial level is necessary to enhance resilience against extreme disruptions.

Fourth, emergency response capabilities should complement network optimization under extreme conditions. When physical connectivity is severely disrupted, internal capacity redundancy alone may be insufficient. Emergency strategies incorporating aerial delivery platforms, mobile medical teams, and cross-regional support mechanisms can provide temporary capacity restoration and maintain essential immunization services in isolated mountainous areas.

### 6.6 Limitations and future work

The framework operates under five scope conditions. First, road-network impedances are measured between county centroids, with intra-county last-mile routing treated as exogenous to the regional model. Second, the model employs a single-period, steady-state planning formulation aligned with routine immunization replenishment cycles: demand reflects monthly immunization volumes and capacity reflects existing static throughput. Shortage behavior under demand surges is evaluated through Scenario B alongside the fixed-topology resilience analysis. Third, equity is operationalized primarily through an economic vulnerability index and spatial accessibility; patient-level access, immunization coverage rates, and downstream health outcomes are left for future examination. Fourth, the model distinguishes planning-stage capacity expansion (unit cost $c_{e}$ ) from emergency expansion (costed at five times $c_{e}$ ), setting aside investment lead times or construction feasibility windows to evaluate stylized capacity scaling. Fifth, uniform transportation cost coefficients are adopted, and network impedances are defined at the regional planning level; future studies could calibrate these cost and capacity parameters with operator-level data.

## 7 Conclusion

This study moves beyond conventional efficiency-oriented logistics by developing a theory-informed optimization framework for vaccine supply networks in complex terrains, conceptually governed by spatial justice and supply chain resilience theories. The framework coordinates three requirements: spatial equity in candidate selection, candidate-pool capacity sufficiency, and network-level survivability. By embedding a vulnerability-adjusted time penalty and a candidate-pool capacity-sufficiency check (α), it jointly incorporates spatial equity and capacity adequacy into candidate selection, ensuring that network optimization proceeds from a pool with adequate aggregate supply capacity.

The empirical results show that the initial 61-hub pool provided aggregate capacity equal to 82% of total demand, and the capacity-sufficiency rule extended the pool to C* (103 hubs) to meet the α = 1.2 requirement. The baseline network achieved zero unmet demand under the endogenous capacity-expansion provision and single-source assignment, and α-sensitivity analysis confirmed that the coefficient provides a transparent planning-stage lever controlling capacity redundancy, expansion relief, and efficiency. Fixed-topology resilience testing further demonstrated that the network sustains zero shortages under structural failures whenever emergency capacity adjustment remains available, whereas zero-expansion stress tests bound shortage-free demand-surge tolerance between +10% and +20% demand growth. These findings indicate that mountainous vaccine supply planning must transcend routine operational efficiency; it requires coordinated candidate-pool adequacy, strategically distributed redundancy, and cross-regional coordination to maintain essential health functions under uncertainty.

## Data Availability

The original contributions presented in the study are included in the article/Supplementary material, further inquiries can be directed to the corresponding author.

## Supplementary material

SUPPLEMENTARY TABLE 1 Detailed routing and branch allocation data for the optimized baseline network.

SUPPLEMENTARY TABLE 2 Node-level capacity expansion, operational status, and emergency expansion quantities under disruption Scenarios A–D.

SUPPLEMENTARY TABLE 3 Mathematical formulation notes, algorithm parameter settings, and supplemental model details.
