# Supplementary material for: Spatial optimization of vaccine supply networks in complex terrains: balancing economic feasibility and public health equity
Source: Front Public Health. 2026 Oct 30;14:1971221. doi: 10.3389/fpubh.2026.1971221 (PMC13759850; doi:10.3389/fpubh.2026.1971221)
Supplement: Supplementary file 1 [file Table_1.DOCX]

Supplementary Material

# 1 Supplementary Tables

Supplementary Table 1. Summary of the optimal two-echelon vaccine distribution network. Characteristics of the 99 operational hubs (selected from 103 candidates) derived from the mixed-integer linear programming (MILP) model. For each operational hub, the table reports the number of served counties, monthly dispatch volume, average branch distance to covered counties, and required capacity expansion. Hubs are ranked in descending order of monthly dispatch volume (total: 1,560,433 doses; total capacity expansion: 186,595 doses). Four candidate hubs with zero allocated flow are omitted. An intra-county branch distance proxy of 5.00 km is assigned to hubs exclusively serving their local jurisdictions to represent baseline last-mile delivery impedance within the county centroid.

| **Supply Hub** | **Covered Counties** | **Monthly Dispatch Volume** | **Avg. Branch Distance (km)** | **Capacity Expansion** |
| --- | --- | --- | --- | --- |
| Shuangliu District | 2 | 65,925 | 14.20 | 9,528 |
| Qingyang District | 5 | 61,694 | 72.23 | 0 |
| Xichang City | 6 | 53,367 | 103.65 | 17,721 |
| Jinniu District | 2 | 50,708 | 12.57 | 0 |
| Shizhong District (Leshan) | 9 | 47,385 | 76.36 | 16,678 |
| Xuzhou District | 5 | 45,536 | 37.67 | 20,096 |
| Fucheng District | 4 | 44,248 | 77.61 | 0 |
| Dongpo District | 6 | 39,024 | 41.37 | 10,574 |
| Wuhou District | 1 | 33,617 | 5.00 | 0 |
| Shunqing District | 4 | 32,656 | 23.84 | 0 |
| Pidu District | 1 | 32,352 | 5.00 | 690 |
| Cuiping District | 3 | 31,138 | 36.21 | 0 |
| Longquanyi District | 1 | 31,091 | 5.00 | 5,027 |
| Bazhou District | 3 | 27,664 | 30.65 | 6,238 |
| Jingyang District | 4 | 26,381 | 40.25 | 0 |
| Guang'an District | 3 | 23,305 | 17.27 | 2,835 |
| Renshou County | 2 | 23,289 | 23.82 | 235 |
| Chuanshan District | 2 | 23,025 | 21.63 | 0 |
| Lizhou District | 4 | 22,638 | 29.65 | 0 |
| Jinjiang District | 2 | 22,612 | 76.28 | 0 |
| Wenjiang District | 1 | 21,975 | 5.00 | 0 |
| Jiangyang District | 3 | 21,612 | 35.16 | 0 |
| Yucheng District | 6 | 21,472 | 60.56 | 3,362 |
| Tongchuan District | 2 | 21,118 | 25.64 | 0 |
| Santai County | 2 | 20,683 | 28.29 | 701 |
| Jianyang City | 1 | 20,444 | 5.00 | 0 |
| Fushun County | 2 | 18,743 | 28.47 | 4,101 |
| Zhaojue County | 2 | 18,641 | 26.70 | 13,876 |
| Xuanhan County | 1 | 18,182 | 5.00 | 1,770 |
| Meigu County | 2 | 17,456 | 70.03 | 12,843 |
| Dongxing District | 2 | 17,217 | 4.02 | 0 |
| Qionglai City | 3 | 17,131 | 46.10 | 2,438 |
| Ziliujing District | 3 | 16,793 | 8.32 | 0 |
| Tongjiang County | 2 | 16,744 | 53.22 | 5,226 |
| Langzhong City | 2 | 16,529 | 16.49 | 1,220 |
| Mianning County | 2 | 16,508 | 35.34 | 9,353 |
| Gulin County | 1 | 16,331 | 5.00 | 4,340 |
| Zhongjiang County | 1 | 14,696 | 5.00 | 0 |
| Jintang County | 1 | 14,689 | 5.00 | 0 |
| Anyue County | 1 | 14,648 | 5.00 | 0 |
| Linshui County | 1 | 14,435 | 5.00 | 337 |
| Nanbu County | 1 | 14,362 | 5.00 | 0 |
| Yanjiang District | 1 | 14,274 | 5.00 | 0 |
| Jiangyou City | 2 | 14,233 | 47.24 | 0 |
| Dazhu County | 1 | 13,594 | 5.00 | 0 |
| Longmatan District | 1 | 13,592 | 6.62 | 0 |
| Qu County | 1 | 13,586 | 5.00 | 0 |
| Pengzhou City | 1 | 13,468 | 5.00 | 0 |
| Yilong County | 1 | 13,410 | 5.00 | 0 |
| Zizhong County | 1 | 13,375 | 5.00 | 0 |
| Dachuan District | 1 | 13,251 | 2.83 | 0 |
| Jialing District | 1 | 13,214 | 5.56 | 5,700 |
| Yuechi County | 1 | 12,777 | 5.00 | 0 |
| Pingchang County | 1 | 12,381 | 5.00 | 0 |
| Dong District | 3 | 12,228 | 9.37 | 0 |
| Lu County | 1 | 11,957 | 5.00 | 0 |
| Dujiangyan City | 1 | 11,917 | 5.00 | 0 |
| Yuexi County | 1 | 11,734 | 5.00 | 5,928 |
| Shifang City | 2 | 11,643 | 15.79 | 0 |
| Shehong City | 1 | 11,581 | 5.00 | 0 |
| Chongzhou City | 1 | 11,539 | 5.00 | 0 |
| Naxi District | 1 | 11,448 | 81.86 | 2,860 |
| Weiyuan County | 2 | 11,385 | 15.51 | 0 |
| Yingshan County | 1 | 10,568 | 5.00 | 0 |
| Hejiang County | 1 | 10,384 | 5.00 | 0 |
| Wusheng County | 1 | 9,709 | 5.00 | 0 |
| Guanghan City | 1 | 9,707 | 5.00 | 0 |
| Miyi County | 2 | 9,610 | 29.09 | 4,252 |
| Leibo County | 1 | 9,311 | 5.00 | 3,794 |
| Longchang City | 1 | 9,177 | 5.00 | 0 |
| Dayi County | 1 | 8,927 | 5.00 | 0 |
| Xindu District | 1 | 8,682 | 13.28 | 0 |
| Huili City | 1 | 8,474 | 5.00 | 3,030 |
| Huidong County | 1 | 7,665 | 5.00 | 275 |
| Chenghua District | 1 | 6,814 | 120.08 | 0 |
| Xinjin District | 1 | 5,253 | 27.50 | 0 |
| Qingbaijiang District | 1 | 4,471 | 139.71 | 0 |
| Litang County | 4 | 4,114 | 166.30 | 2,466 |
| Mao County | 3 | 3,985 | 88.77 | 747 |
| Shiqu County | 1 | 3,766 | 5.00 | 2,462 |
| Kangding City | 2 | 3,440 | 100.96 | 0 |
| Dege County | 1 | 2,899 | 5.00 | 1,458 |
| Aba County | 1 | 2,406 | 5.00 | 561 |
| Wenchuan County | 2 | 2,149 | 30.65 | 0 |
| Seda County | 1 | 2,144 | 5.00 | 855 |
| Ruoergai County | 1 | 2,082 | 5.00 | 305 |
| Ganzi County | 1 | 2,060 | 5.00 | 480 |
| Ma'erkang City | 2 | 2,037 | 44.97 | 0 |
| Xiaojin County | 2 | 1,892 | 32.49 | 0 |
| Luding County | 1 | 1,691 | 5.00 | 0 |
| Rangtang County | 1 | 1,604 | 5.00 | 596 |
| Baiyu County | 1 | 1,478 | 5.00 | 257 |
| Jiuzhaigou County | 1 | 1,448 | 5.00 | 0 |
| Luhuo County | 1 | 1,406 | 5.00 | 230 |
| Hongyuan County | 1 | 1,398 | 5.00 | 367 |
| Xinlong County | 1 | 1,361 | 5.00 | 186 |
| Batang County | 1 | 1,254 | 5.00 | 218 |
| Jiulong County | 1 | 1,243 | 5.00 | 242 |
| Yajiang County | 1 | 1,173 | 5.00 | 139 |

**Supplementary Table 2. Detailed scenario-level results of the resilience stress tests reported in Tables 2 and 3 of the main text, including failed-node identities and emergency-expansion volumes under the expansion-capable and zero-expansion settings.**

| **Setting** | **Scenario** | **Demand multiplier** | **Objective Z (model cost units)** | **Total Shortage (doses)** | **Failed Nodes** | **Emergency Expansion (doses)** | **Cost Growth (%)** |
| --- | --- | --- | --- | --- | --- | --- | --- |
| Expansion-capable | Baseline | 1.0x | 18,510,357 | 0 | None | 0 | 0.00% |
| Expansion-capable | Scenario A (peripheral failure) | 1.3x | 24,624,444 | 0 | Rangtang County; Kangding City | 231,900 | 33.03% |
| Expansion-capable | Scenario B (+10% demand) | 1.1x | 20,446,371 | 0 | None | 58,627 | 10.46% |
| Expansion-capable | Scenario B (+30% demand) | 1.3x | 24,603,126 | 0 | None | 227,701 | 32.92% |
| Expansion-capable | Scenario B (+50% demand) | 1.5x | 28,957,877 | 0 | None | 480,010 | 56.44% |
| Expansion-capable | Scenario B (+70% demand) | 1.7x | 34,498,697 | 0 | None | 750,700 | 86.38% |
| Expansion-capable | Scenario C (regional multi-hub failure) | 1.3x | 25,352,322 | 0 | Qingyang District; Xichang City; Jinniu District | 366,215 | 36.96% |
| Expansion-capable | Scenario D (largest-hub failure) | 1.3x | 24,759,454 | 0 | Shuangliu District | 298,552 | 33.76% |
| Zero-expansion | Baseline | 1.0x | 18,417,060 | 0 | None | 0 | 0.00% |
| Zero-expansion | Scenario B (+10% demand) | 1.1x | 21,884,614 | 0 | None | 0 | 18.83% |
| Zero-expansion | Scenario B (+20% demand) | 1.2x | 124,985,289 | 71,308 | None | 0 | 578.64% |
| Zero-expansion | Scenario B (+30% demand) | 1.3x | 428,930,827 | 203,084 | None | 0 | 2,228.99% |
| Zero-expansion | Scenario A (peripheral failure) | 1.0x | 19,123,683 | 0 | Rangtang County; Kangding City | 0 | 3.84% |
| Zero-expansion | Scenario C (regional multi-hub failure) | 1.0x | 19,911,965 | 0 | Qingyang District; Xichang City; Jinniu District | 0 | 8.12% |
| Zero-expansion | Scenario D (largest-hub failure) | 1.0x | 19,156,634 | 0 | Shuangliu District | 0 | 4.02% |

**Note:** Cost growth is calculated relative to setting-specific baselines: 18,510,357 for expansion-capable scenarios and 18,417,060 for zero-expansion scenarios (excluding routine expansion investment). Demand levels are scaled to 1.3x in expansion-capable Scenarios A, C, and D, and set to 1.0x in corresponding zero-expansion failure scenarios. Fixed-topology tests retain the baseline hub network, remove failed facilities, and reoptimize demand allocation across operational hubs. A disconnection penalty ($M_{p}={10}^{8}$) is applied per fully isolated county to ensure full network connectivity, meaning reported shortages stem strictly from binding capacity constraints. Costs and shortage quantities are rounded to the nearest integer, and all doses denote monthly volumes.
